# Long-Term Immunological Memory of SARS-CoV-2 Is Present in Patients with Primary Antibody Deficiencies for up to a Year after Vaccination

**DOI:** 10.3390/vaccines11020354

**Published:** 2023-02-03

**Authors:** Zane Lucane, Baiba Slisere, Lota Ozola, Dmitrijs Rots, Sindija Papirte, Baiba Vilne, Linda Gailite, Natalja Kurjane

**Affiliations:** 1 Riga Stradins University, Riga LV-1007, Latvia; 2 Pauls Stradins Clinical University Hospital, Riga LV-1007, Latvia; 3 Children’s Clinical University Hospital, Riga LV-1007, Latvia

**Keywords:** inborn error of immunity, antibody deficiency, common variable immunodeficiency, selective IgA deficiency, SARS-CoV-2, immune response, T-cell response, antibody response

## Abstract

Some studies have found increased coronavirus disease-19 (COVID-19)-related morbidity and mortality in patients with primary antibody deficiencies. Immunization against COVID-19 may, therefore, be particularly important in these patients. However, the durability of the immune response remains unclear in such patients. In this study, we evaluated the cellular and humoral response to severe acute respiratory syndrome coronavirus 2 (SARS-CoV-2) antigens in a cross-sectional study of 32 patients with primary antibody deficiency (*n* = 17 with common variable immunodeficiency (CVID) and *n* = 15 with selective IgA deficiency) and 15 healthy controls. Serological and cellular responses were determined using enzyme-linked immunosorbent assay and interferon-gamma release assays. The subsets of B and T lymphocytes were measured using flow cytometry. Of the 32 patients, 28 had completed the vaccination regimen with a median time after vaccination of 173 days (IQR = 142): 27 patients showed a positive spike-peptide-specific antibody response, and 26 patients showed a positive spike-peptide-specific T-cell response. The median level of antibody response in CVID patients (5.47 ratio (IQR = 4.08)) was lower compared to healthy controls (9.43 ratio (IQR = 2.13)). No difference in anti-spike T-cell response was found between the groups. The results of this study indicate that markers of the sustained SARS-CoV-2 spike-specific immune response are detectable several months after vaccination in patients with primary antibody deficiencies comparable to controls.

## 1. Introduction

Primary antibody deficiencies (PAD) are a group of rare diseases that account for about 55% of all inborn errors of immunity (IEI) [1]. Selective immunoglobulin A (IgA) deficiency (sIgAD) is the most common PAD. It is characterized by diminished serum IgA levels with normal IgM and IgG levels and is often asymptomatic but may present with recurrent infections or immune dysregulation-related symptoms such as autoimmunity or atopic diseases [2]. The most common symptomatic IEI is common variable immunodeficiency (CVID), characterized by reduced serum IgG and IgA and/or IgM levels and poor vaccination responses. CVID is manifested by severe and recurrent infections, and up to 66% present with various non-infectious complications, such as autoimmunity, enteropathy, and malignancy [3].

In late 2019, a new coronavirus, severe acute respiratory syndrome coronavirus 2 (SARS-CoV-2), was identified [4], causing a pandemic of acute respiratory syndrome known as coronavirus disease-19 (COVID-19) with significant morbidity and mortality in the general population [5]. Early reports showed an increased risk of severe COVID-19 disease in subjects with immunosuppression [6]. Therefore, patients with inborn errors of immunity were of special interest, as a defect in the immune system may predispose them to severe infections, including severe COVID-19, especially in patients with defects in type I interferon signaling pathways [7]. On the other hand, immunosuppression could reduce immune responses, cytokine storms, and inflammatory processes, constituting a protective factor against COVID-19 [8]. Although some studies concluded that for the majority of patients, the underlying IEI was not an independent risk factor for severe COVID-19 [9,10,11,12], some studies found increased morbidity and/or mortality, including in patients with common variable immunodeficiency [13,14,15]. SIgAD has also been identified as a risk factor for a more severe COVID-19 course [16,17]. Therefore, vaccination against COVID-19 may be particularly important in IEI patients. The European Society for Immunodeficiencies (ESID) recommends that patients with IEI receive any of the available COVID-19 vaccines according to the national vaccination schedule that includes a primary vaccination course and booster doses [18].

Overall, the long-term durability of the antibody response is poorly understood, with several studies showing a trend toward decreasing antibody levels over time in immunocompetent individuals [19,20,21,22] while the receptor-binding domain (RBD) of the spike protein-specific memory B-cell fraction persists [23]. This trend of decrease in antibody levels has also been observed 6 months after the vaccination in primary antibody deficiency patients [24]. Furthermore, a limited neutralizing capacity of anti-spike SARS-CoV-2 antibodies has been reported in these patients [25,26]. Therefore, since the mechanisms of somatic mutation and selection in germinal centers that lead to differentiation of mature class-switched memory B cells and antibody response may be altered in patients with specific antibody deficiencies [27], several studies have highlighted the importance of assessing cellular responses specific for the SARS-CoV-2 antigen, when evaluating vaccine-induced immunity in patients with IEI [28]. Even in cases where the patient does not develop a humoral immune response to COVID-19 at a protective level, the vaccine could still be highly effective, as the presence of memory T cells can help control the infection. The role of T cells in COVID-19 protection is supported by reports of a lack of SARS-CoV-2 responsive CD4+ T cells in severely infected patients, as well as a milder disease in patients with early induction of functional SARS-CoV-2-specific T cells, or the presence of T-cell responses without a humoral response in asymptomatic individuals [29,30,31]. The durability of SARS-CoV-2 specific T-cell responses could also be higher than the durability of humoral responses [32]; however, the long-term SARS-CoV-2-specific T-cell memory in patients with antibody deficiency remains unclear. In addition, no study to date has investigated the humoral and cellular immune response to SARS-CoV-2 vaccination, specifically in sIgAD patients. In addition, only a few studies have explored immunological correlations and predictive markers associated with higher immune responses after SARS-CoV-2 vaccination, most of them evaluating early immune responses [28,33,34,35,36].

The primary objective of this study was to evaluate a long-term SARS-CoV-2 spike-specific humoral and cellular immune response in adult patients with the most common types of primary antibody deficiency (sIgAD and CVID) compared to healthy controls of the same age. The secondary objective was to identify predictive markers that are associated with a better immune response after COVID-19 vaccination in the patient group.

## 2. Materials and Methods

### 2.1. Subjects

Patients with CVID and symptomatic sIgAD who were treated at a tertiary immunology center (Pauls Stradins Clinical University Hospital, Riga, Latvia) were invited to participate in the study from April to July 2022. A total of 47 subjects were recruited for the study, including 17 patients with CVID, 15 patients with symptomatic sIgAD, and 15 healthy controls. The diagnosis was based on diagnostic criteria of the European Society for Immunodeficiencies (ESID) clinical diagnostic criteria [37]. All patients were re-evaluated to meet the diagnostic criteria of CVID. Relevant clinical data on CVID and sIgAD patients were obtained from patient electronic health records, including data on patient age, sex, family history of inborn error of immunity, treatment, history of SARS-CoV-2 vaccination and infection, confirmed by a positive polymerase chain reaction (PCR), clinical characteristics, including prior infections, infectious complications, such as conductive hearing loss and bronchiectasis and non-infectious complications, such as autoimmunity, polyclonal benign lymphoproliferation, granulomatous disease, enteropathy, and malignancy. A SARS-CoV-2 vaccination course was considered complete if the patient received one dose after recovery from COVID-19 or two doses if not previously infected with SARS-CoV-2. The severity of COVID-19 in the personal history was assessed using the World Health Organization (WHO) clinical progression scale [38]. Patients who were unvaccinated were excluded from association analyses. Blood samples were obtained from patients and controls. All subjects provided written consent to participate in this study. This study was performed in accordance with the principles of the Declaration of Helsinki. The study protocol was reviewed and approved by the Central Board of the Ethics Committee of the Health Ministry of the Republic of Latvia (No. 01–29.1/2878).

### 2.2. Antibody Response to SARS-CoV-2

Serum separator tubes were used to separate serum by centrifugation. Serum samples were tested using the commercial, semi-quantitative enzyme-linked immunosorbent assay (ELISA) that detects specific SARS-CoV-2- IgG antibodies against the S1 domain of the spike protein (Euroimmun Anti-SARS-CoV-2 IgG assay, Euroimmun, Lübeck, Germany), following the manufacturer’s recommendations. An IgGAM ratio (optical density compared with calibrator) > 1.1 was considered a positive response.

### 2.3. T-Cell Response to SARS-CoV-2

To detect the response of SARS-CoV-2 spike protein-reactive T cells, an interferon-gamma release assay (IGRA) QuantiFERON SARS-CoV-2 (Qiagen, Hilden, Germany) was used. The SARS-CoV-2 spike (S) protein consists of a signal peptide, an N-terminal S1 protease fragment (also containing receptor-binding domain (RBD)), and a C-terminal S2 protease fragment [29]. Specialized QuantiFERON starter set blood collection tubes were used to collect blood: Ag1 tube, containing T-cell epitopes within the receptor-binding domain of S1 (measures CD4+ T-cell responses); Ag2 tube, containing T-cell epitopes within S1 and S2 (measures CD4+ and CD8+ T-cell responses), as well as positive and negative control tubes. The tubes were incubated at 37 °C for 20 h, then centrifuged for plasma separation and froze at −20 °C for further analysis of interferon-gamma (IFN-γ) production using an ELISA, as previously reported [39], according to the manufacturer’s protocol. A value >0.15 IU/mL was considered a positive response.

### 2.4. Lymphocyte Isolation and Flow Cytometry

Fresh blood samples were collected in tubes containing lithium heparin. Briefly, heparinized whole blood samples were diluted and transferred to SepMate peripheral blood mononuclear cell (PBMC) isolation tubes (STEMCELL Technologies, Vancouver, Canada) containing Histopaque-1077 density gradient cell separation medium (Sigma-Aldrich, Saint Louis, MO, USA) and isolated following the manufacturer’s instructions. The viability of PBMCs was detected by trypan blue staining.

After washing with phosphate-buffered saline (Thermo Fisher Scientific, Waltham, MA, USA) with 2% fetal bovine serum (Sigma Aldrich, Saint Louis, MO, USA), isolated PBMCs were incubated in 96-well plates with a fragment crystallizable region (Fc) blocking reagent (Miltenyi Biotec, Bergisch Gladbach, Germany). The PBMCs were then stained with a mixture of the following antibodies at optimal concentrations: anti-21 fluorescein isothiocyanate (FITC), anti-CD27 phycoerythrin (PE)/Dazzle594, anti-CD268 peridinin chlorophyll protein (PerCP)/Cyanine5.5 (Cy5.5), IgD-PE/Cyanine7 (Cy7), anti-IgM allophycocyanin (APC) (all from BioLegend, San Diego, CA, USA), anti-CD19 Alexa Fluor 700 (A700) (from BD Biosciences, San Jose, CA, USA) anti-CD38 PE (from Beckman Coulter, Brea, CA, USA) for B-cell panel; anti-CD45RA electron-coupled dye (ECD), anti-CD45RO PE/Cy5.5, anti-CD8 APC/A700 (all from Beckman Coulter, Brea, CA, USA), anti-CD27 PE/Cyanine7, anti-CD4 APC, anti-CD3 APC/Cy7 (all from BioLegend, San Diego, CA, USA) for T-cell panel; anti-CD25 PE, anti-CD127 PE/Cy7, anti-CD4 APC, anti-CD8 APC/A700, anti-CD3 APC/Cy7 (all from BioLegend, San Diego, CA, USA) for T regulatory cell panel. The PBMCs were washed and then fixed with formaldehyde. For the staining of the nuclear FOXP3 antigen, anti-FOXP3-Alexa Fluor 488 (A488) (BioLegend, San Diego, CA, USA) and the Transcription Factor Staining Buffer Set (BD Biosciences, San Jose, CA, USA) were used according to the manufacturer’s instructions, adjusted for 96-well staining. Data were obtained as LMD files using a Beckman Coulter Navios Ex flow cytometer and analyzed using Kaluza 2.1 software (Beckman Coulter, Brea, CA, USA). Manual gating was applied, as shown in Supplementary Material S1.

B cells were subdivided into the following subpopulations: naïve B cells (CD19+CD27−IgM+IgD+), marginal zone B cells (CD19+CD27+IgM++IgD+), switched memory B cells (CD19+CD27+IgM−IgD−), IgM-only memory B cells (CD19+CD27+IgM++IgD−), transitional B cells (CD19+IgD+CD27-IgM++CD38++), CD21low B cell (CD19+ IgM+, CD21-CD38-), plasmablasts (CD19+CD21+CD38+++IgM−), atypical memory B cells (CD19+CD21-CD27-IgD-). T cells were subdivided as follows: naïve T helper cells (CD3+CD4+CD27+CD45RA+), central/transitory memory T helper cells (CD3+CD4+CD27+CD45RA-), effector memory T helper cells (CD3+CD4+CD27-CD45RA-), terminally differentiated T helper cells (CD3+CD4+CD27-CD45RA+), recent thymic emigrant T cells (CD3+CD4+CD31+CD45RO-), naïve T cytotoxic cells (CD3+CD8+CD27+CD45RA+), central/transitory memory T cytotoxic cells (CD3+CD8+CD27+CD45RA-), effector memory T cytotoxic cells (CD3+CD8+CD27-CD45RA-), terminally differentiated T cytotoxic cells (CD3+CD8+CD27-CD45RA+). T regulatory cells were identified based on the following parameters: CD3+CD4+CD25+FOXP3+CD127dim.

### 2.5. Clinical and Immunologic Phenotyping of CVID Patients

Clinical phenotyping was based on the classification suggested by Chapel et al. [40] and included patients with infection-only, autoimmunity, enteropathy, polyclonal lymphocytic infiltration, malignancy, and overlapped phenotype (if the patient had more than one non-infectious complication). The severity of CVID was assessed using CVID Severity Score proposed by Ameratunga [41]. Immunological phenotyping was based on the most commonly used classifications of B-cell subtypes in CVID patients: Paris [42], Freiburg [43], EUROclass [44], and B-cell pattern classifications [45].

### 2.6. Data Analysis

The normality of the data was assessed using the Shapiro–Wilk test. The results indicated that the data were not normally distributed; therefore, nonparametric statistical methods were used in subsequent analysis. Differences in categorical variables were examined by using chi-square and Fisher exact tests. The Mann–Whitney U or Kruskal–Wallis tests were used to compare continuous variables by two or more groups, respectively. Spearman’s rank test was used to assess the correlation between continuous variables. A *p*-value < 0.05 was considered statistically significant. Statistical analysis was performed using IBM SPSS Statistics Version 23 (IBM, New York, NY, USA). Graphs were generated using GraphPad Prism 8 (GraphPad Software, San Diego, CA, USA).

## 3. Results

### 3.1. Subjects

A total of 32 patients with primary antibody deficiencies (aged 38.5 (IQR = 21) years) and 15 healthy vaccinated controls (aged 37 (IQR = 19) years) were enrolled in the study. Table 1 summarizes the demographic and clinical data of patients and controls.

Regarding treatment, in the CVID group, all patients except two received regular immunoglobulin replacement therapy (subcutaneously 100 mg/kg/week). A patient with sIgAD with rheumatoid arthritis was on regular immunosuppressive treatment with corticosteroids (methylprednisolone 8 mg/day), methotrexate 15 mg/week, and biological therapy with JAK inhibitor (upadacitinibum 15 mg/day). Two patients had previously received chemotherapy for neoplasia, and one patient was treated with rituximab (the last dose was administered 6 months prior to vaccination).

### 3.2. Vaccination Status and Previous SARS-CoV-2 Infection

Of the 32 patients enrolled in this study, 28 had completed the SARS-CoV-2 immunization schedule with the messenger ribonucleic acid (mRNA) vaccine (27 patients) or the adenovirus vector vaccine (1 patient) before enrollment in a study with a median time of 173 days (IQR = 142) after the last vaccine dose, ranging from 25 to 345 days (see Table 2). All healthy controls were vaccinated with mRNA vaccines, with days after the last dose ranging from 96 to 511 (median 215, IQR = 201).

Four patients refused the vaccination but had previously had COVID-19 in their personal medical history. None of these four patients showed a positive T-cell response to the SARS-CoV-2 S1/S2 pool antigens. Two of these patients had CVID, and two—sIgAD. Two patients with the most recent SARS-CoV-2 infection—a CVID patient 58 days after positive SARS-CoV-2 PCR and a selective IgA patient 108 days after positive PCR—showed a positive anti-spike humoral response (anti-spike IgG antibody ratio 9.91 and 2.2, respectively). The remaining two unvaccinated patients had a positive SARS-CoV-2 PCR test about a year ago, and their serum antibody levels were undetectable. We further analyzed vaccinated individuals only.

### 3.3. Humoral and Cellular Response to SARS-CoV-2 Vaccine

**Figure 1:**
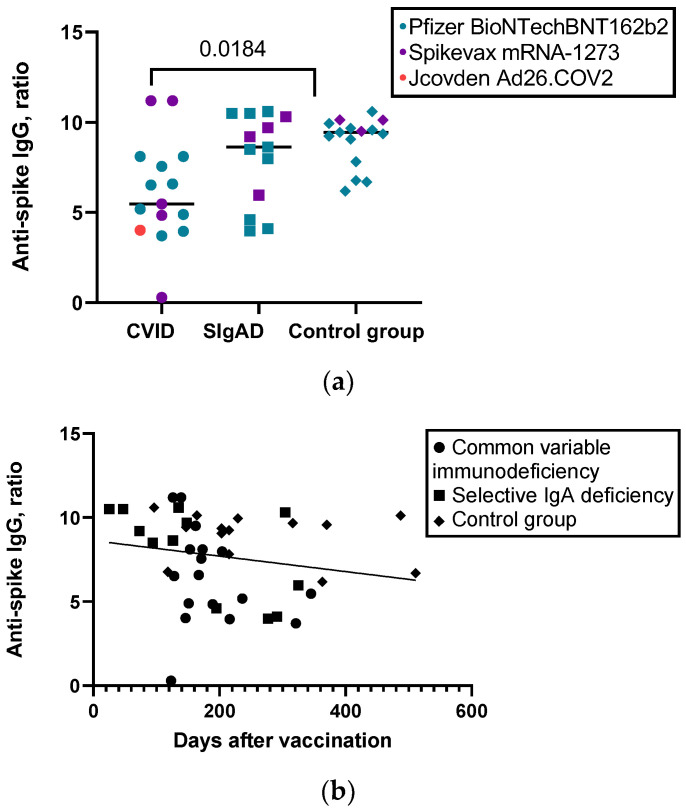
(**a**) Levels of anti-spike IgG antibody levels in different groups; (**b**) correlation between anti-spike IgG antibody levels and days after vaccination.

Overall, all vaccinated patients with sIgAD had anti-spike IgG antibody levels >1.1 ratios (see Table 2). Of the 15 vaccinated patients with CVID, 14 tested positive for anti-spike IgG antibodies (including the patient on rituximab), showing a wide range of antibody levels, from undetectable levels to normal or high titers. However, as can be seen in Figure 1a, the median level of antibody response in CVID patients was lower compared to the healthy controls. Overall, there was a significant but weak negative correlation between antibody levels and days after vaccination (r_s_ = −0.302; *p* = 0.049; see Figure 1b). This correlation was also observed in the sIgAD subgroup (r_s_ = −0.247, *p* = 0.038) but was not present in the CVID subgroup (r_s_ = −0.247; *p* = 0.376) and in healthy controls (r_s_ = −0.182; *p* = 0.515).

**Figure 2:**
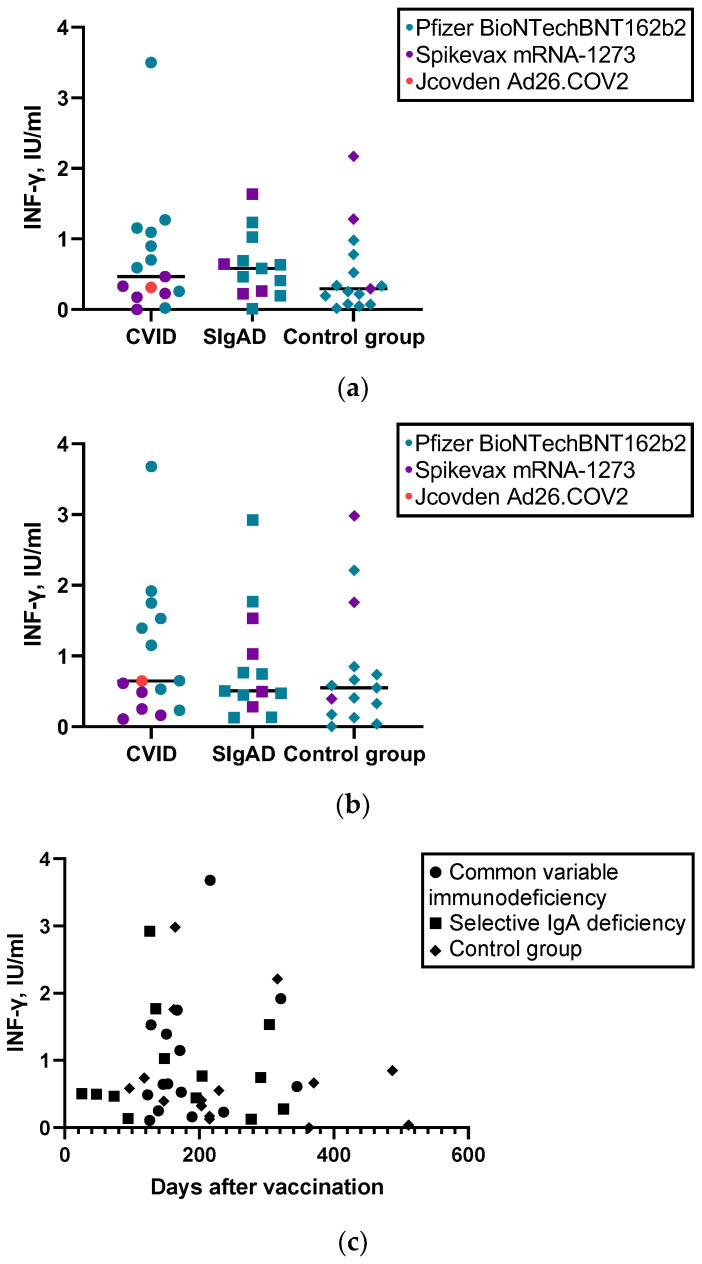
(**a**) CD4+ cell response (levels of INF-y) to S1 pool-specific protein in different groups; (**b**) CD4+ and CD8+ cell response (levels of INF-y) to S1/S2 pool-specific protein in different groups; (**c**) Correlation between anti-spike CD4+ and CD8+ cell response and days after vaccination.

Of the 15 CVID-vaccinated patients, 14 showed a positive anti-spike T-cell response (see Table 2). The only non-responsive CVID patient had three autoimmune diseases (type 1 diabetes, celiac disease, chronic autoimmune thyroiditis) and a baseline serum INF-γ level of 3.06 IU/mL and exhibited high anti-spike IgG antibody titers (11.2 ratio). All patients with sIgAD, except for the patient on immunosuppressive therapy, had positive anti-spike T-cell response. Evaluation of T-cell responses showed no significant differences in spike-specific IFN-γ production between the study groups, as well as no correlation with days after vaccination (see Figure 2).

Interestingly, regarding healthy controls, three healthy controls did not exhibit an anti-spike T-cell response (on days 215; 363; 511 after the last vaccine dose) but showed a positive humoral response (anti-spike IgG antibody ratio 9.23, 6.18, and 6.69, respectively). Furthermore, we observed a positive correlation between anti-spike IgG antibody levels and T-cell response (INF-y synthesis to the S1 and S2 pools) in the control group (S1 pool (r_s_ = 0.571; *p* = 0.026) and to the S1/S2 pool (r_s_ = 0.721; *p* = 0.02)), but we did not observe this trend in the sIgAD or CVID groups.

### 3.4. Immunological Memory to SARS-CoV-2 and Demographic/Clinical/Immunologic Phenotyping Markers

Age did not significantly influence the extent of humoral or cell response in any of the groups. We did not observe any differences in humoral or T-cell anti-spike responses between COVID-19-convalescent individuals and subjects naïve to infection in any of the groups.

No differences in humoral or T-cell immune responses were associated with the patient’s clinical parameters, except in the sIgAD group, patients with recurrent otitis had a statistically significantly lower median T-cell response to S1/S2 antigen compared to patients without this infection (*p* = 0.038; median 0.5 (IQR = 0.8) and 0.13, respectively). In CVID patients, no correlation was found between humoral or T-cell response and the Ameratunga CVID severity score. We did not observe any difference in humoral or T-cell response between different groups of CVID Chapel phenotypes.

A significant correlation was found between the anti-spike antibody response and central memory CD8+ cell percentages (r_s_ = 0.385; *p* = 0.047), and in the selective IgA patient group also, central memory CD4+ cell percentages (r_s_ = 0.635; *p* = 0.020). We found no significant relationship between the anti-spike cellular response and the subsets of B cells. No significant correlation was observed between anti-spike IgG antibody or T-cell response and total IgG, IgM, or IgA levels.

In CVID patients, immunological classification of the patients did not reveal a difference in anti-spike humoral or T-cell response between different immunological classifications according to Paris, Freiburg, EUROclass, or B-cell pattern classifications. The only B-cell-negative CVID patient (group B- according to EUROclass classification) also exhibited a positive anti-spike IgG antibody level (11.2 ratio) and anti-spike T-cell response (0.25 IU/mL) 139 days after concluding the primary vaccination regimen with the mRNA-1273 vaccine.

## 4. Discussion

In this study, we evaluated a long-term SARS-CoV-2 spike-specific humoral and cellular immune response in a cohort of 32 adult patients with CVID (which represents 85% of patients diagnosed with CVID in Latvia [46]) or symptomatic sIgAD and 15 healthy controls. Our data suggest that CVID and sIgAD patients show a humoral and cellular immune response to the SARS-CoV-2 vaccine that is present several months after vaccination, but the detectable anti-spike IgG antibody levels in CVID patients are lower than in healthy controls, and selective IgA patients.

A previous report examining the early response to vaccination had revealed positive anti-spike antibodies with a wide variation in seropositivity rates after vaccination, ranging between 20.6% and 90.9% [25,26,28,33,34,35,47,48] and cellular response in up to 85% of patients tested with IEI [35]. In our cohort, we observed comparable rates of detectable anti-spike antibodies (93% in CVID patients) and anti-spike T-cell responses (93% in CVID patients). However, almost all CVID patients received subcutaneous immunoglobulin (SCIG) replacement therapy, and this should be taken into consideration when evaluating humoral responses, as it is likely that the pool of immunoglobulin preparations contains specific antibodies to SARS-CoV-2 [49,50]. In contrast, earlier findings of Pham suggested that intravenous immune globulin (IVIG) preparations received in September 2021 did not significantly alter patient levels of SARS-CoV-2 antibodies [25]. In addition, we did not observe a correlation between total IgG and spike-specific IgG antibody levels.

We demonstrated that patients with CVID and sIgAD are capable of generating an anti-spike T-cell response at least up to almost a year (up to 345 days) after the completion of the vaccination schedule, consistent with the relatively sparing T-cell immunity of these patients. The results are in line with previously described that contrary to protein vaccines, mRNA vaccine formulations also trigger robust CD4+ T-cell responses and strong CD8+ T-cell responses, perhaps as a result of the effective presentation of endogenously generated antigens on major histocompatibility complex (MHC) class I molecules [51]. Similar to our findings, other studies that measured early vaccine responses also found no statistically significant differences in anti-spike T-cell response between healthy vaccinated controls and patients with CVID or sIgAD [33]. On the contrary, in IEI patients, a significantly higher subset of SARS-CoV-2 antigen-specific CD4 + CD40L + T cells have been described a month after vaccination, suggesting T-cell compensatory function in patients with primary B-cell impairment [28]. However, these different outcomes could be explained by differences in the methodology, as it is uncertain how much the various assays (cytokine production, antigen-induced proliferative responses, or the overexpression of certain activation markers) accurately represent the same characteristics of a T-cell response, and there is no clear consensus on methods of how to identify vaccine-specific T-cell responses [52].

Studies with healthy donors have found a positive correlation between the magnitude of anti-spike CD4+ T cells with anti-spike IgG antibody responses early after the vaccination and, consistent with the concept of intramolecular help, also anti-spike CD8+ T-cell responses, suggesting the concurrent development of adaptive humoral and cellular immunity [29]. However, studies on long-term immunological memory in healthy individuals have also found antibody titers not to be predictive of T-cell memory [53]. We found a relationship between anti-spike humoral and cellular responses in the control group; however, this link was not observed in the CVID group and, interestingly, in the selective IgA patient group who did not receive immunoglobulin replacement therapy.

Older people have been reported to be at increased risk of severe COVID-19 infection. Although there are several factors related to the increased risk, one of these risk factors could be a reduced T-cell response, partly due to a more limited repertoire of naïve T cells [31]. It has been described that the immune response to vaccination is often also weaker, with a limited duration of protection in elderly individuals [54]. This trend has also been documented by Hagin et al. in CVID patients [33]. However, we did not observe an association between the humoral or cellular immune response to vaccination and age in our cohort of primary antibody patients, similar to other reports [25,26,28].

Although it has previously been described that the humoral and T-cell response of patients with previous SARS-CoV-2 infections was significantly greater than that of patients with infection-naïve individuals after vaccination [26,34], we did not observe this in our cohort. However, these differences have been suggested to arise from constituents of the peptide pool in interferon-gamma release assays (IGRA) tests [26].

Primary antibody deficiency patients with a history of autoimmunity have previously been reported to have a poorer response to vaccination [36]. In this study, we were not able to identify an association between the magnitude of the immune response and clinical manifestations. Regarding immunological correlates with vaccine responsiveness, we found a significant correlation between the anti-spike humoral response and central memory CD8+ cell percentages and in the selective IgA patient group—also with central memory CD4+ cell percentages. The results are consistent with a previously reported expansion of the T-cell memory subsets in patients with IEI and healthy controls 28 days after vaccination with the BNT162b2 mRNA vaccine [28]. However, a recent study examining immunological predictors of impaired immune response to SARS-CoV-2 vaccination found no association to central memory CD4+ or CD8+ T cells, but a significantly higher frequency of effector memory CD8+ T cells in those of impaired humoral immune response [36], and a similar trend has also been observed with humoral response to influenzae vaccination in CVID patients [55]. We found no significant relationship between long-term anti-spike humoral response and B-cell subsets, consistent with reports on early vaccination responses in IEI patients where there was no variation in the B-cell compartment a month after vaccination was reported [28]. However, in PAD patients, lower levels of baseline IgG, IgA, total B cells, and switched memory B cells were related to poor SARS-CoV-2 vaccine anti-spike antibody response [36]. In addition, following influenza vaccination, in patients with CVID, numbers of circulating switched memory B lymphocytes (EUROClass B+ smB+) were directly correlated with a superior humoral immune response [56].

Several major limitations of this study should be considered. First, the small sample size. Second, the observational design of the study and the broad time frame within the measurements after completion of the immunization schedule does not allow us to conclude if the presence of anti-spike antibodies or anti-spike T-cell responses can confer protection against different SARS-CoV-2 or to evaluate the dynamics of the immune responses. Third, we did not include the evaluation of SARS-CoV-2-specific memory B cells in our study. Regarding long-term immunological memory in healthy patients, a trend of increased SARS-CoV-2-specific memory B cells several months after infection in healthy individuals reconvalescent from SARS-CoV-2 [53]. In patients with antibody deficiencies, Salinas et al. found an impaired spike-specific memory B-cell compartment in CVID patients, compared to healthy controls after vaccination with Pfizer/BioNTech. They also found a higher frequency of spike-specific atypical memory B-cell subsets in CVID patients compared to healthy controls after vaccination, suggesting that they retreated from extrafollicular reactions rather than germinal center reactions [34].

In conclusion, this study provides information on long-term immunological memory in patients with CVID and sIgAD. Our findings confirm that markers of the sustained SARS-CoV-2 spike-specific humoral and cellular immune response are detectable several months after vaccination. Despite the significantly lower median levels of anti-spike IgG response in CVID patients than in healthy controls, the T-cell response in CVID and sIgAD patients was comparable to that of healthy controls; therefore, vaccination should be recommended in these patients.

## Figures and Tables

**Table 1:** Demographic and clinical parameters of the patients and controls.

Parameters	CVID	sIgAD	Healthy Controls	*p*
Number	17	15	15	-
Sex				
Female, *n* (%)	10 (58.8%)	12 (80%)	10 (66.6%)	0.460
Male, *n* (%)	7 (41.2%)	3 (20%)	5 (33.4%)	
Age, median (IQR)	40 (24)	37 (21)	37 (19)	0.467
Ethnicity, Caucasian, *n*	17 (100%)	15 (100%)	15 (100%)	-
Age at diagnosis, median (IQR)	34 (28)	33 (45)		0.664
Positive family history, *n* (%)	1 (5.8%)	1 (6.6%)		0.755
Clinical characteristics				
Recurrent infections, *n* (%)	17 (100%)	13 (86.6%)		
Recurrent pneumonia, *n* (%)	15 (88.2%)	1 (6.6%)		
Recurrent otitis media, *n* (%)	8 (47%)	2 (13.3%)		
Recurrent sinusitis, *n* (%)	12 (70.5%)	3 (20%)		
Recurrent urinary tract infections, *n* (%)	2 (11.7%)	3 (20%)		
Sepsis in personal medical history, *n* (%)	0 (0%)	1 (6.6%)		
Bronchiectasis, *n* (%)	6 (35.3%)	0 (0%)		
Conductive hearing impairment, *n* (%)	3 (17.6%)	0 (0%)		
Autoimmunity, *n* (%)	6 (35.3%)	8 (53.3%)		0.784
Splenomegaly, *n* (%)	5 (29.4%)	1 (6.7%)		0.178
Hepatomegaly, *n* (%)	3 (17.6%)	1 (6.7%)		0.603
Enteropathy, *n* (%)	4 (23.5%)	3 (20.0%)		0.576
Malignancy, *n* (%)	2 (11.8%)	1 (6.7%)		0.548
Allergy or atopy, *n* (%)	10 (58.8%)	8 (53.3%)		0.517
CVID severity score, median	15 points (IQR = 19)			
Positive SARS-CoV-2 PCR in personal medical history, *n* (%)	10 (58.8%)	9 (60.0%)	8 (53.8%)	0.918
Severity of COVID-19 according to WHO clinical progression scale *				
Asymptomatic (score 1), *n*	1	0	0	
Mild (not hospitalized 2–3), *n*	6	8	8	
Moderate (hospitalized 4–5), *n*	2	1	0	
Severe (hospitalized 6 + ), *n*	1	0	0	

Abbreviation: CVID—common variable immunodeficiency; sIgAD—selective IgA deficiency; *n*—number; IQR—interquartile range; SARS-CoV-2—severe acute respiratory syndrome coronavirus 2; PCR—polymerase chain reaction; COVID-19—coronavirus disease 2019; WHO—World Health Organization. *p*-values of less than 0.05 were regarded as significant. * Ambulatory mild disease: score 1—asymptomatic, viral ribonucleic acid (RNA) detected; score 2—symptomatic—independent; score 3—symptomatic, assistance needed; Hospitalized: moderate disease: score 4—hospitalized, no oxygen therapy required; score 5—hospitalized, oxygen by mask or nasal prongs; Hospitalized: severe disease: score 6—hospitalized with non-invasive ventilation or high flow oxygen; score 7—intubation and mechanic ventilation partial pressure of oxygen/fraction of inspired oxygen ratio (pO_2_/FiO_2_) ≥ 150 or oxygen saturation/fraction of inspired oxygen ratio (SpO_2_/FiO_2_) ≥ 200; score 8—mechanic ventilation pO_2_/FiO_2_ < 150 or SpO_2_/ FiO_2_ < 200 or vasopressors; score 9—mechanic ventilation pO_2_/FiO_2_ < 150 or SpO_2_/ FiO_2_ < 200 and vasopressors, dialysis or extracorporeal membrane oxygenation (ECMO); Dead: score 10—dead.

**Table 2:** Vaccination-related parameters in patients and controls.

Parameter	CVID	sIgAD	Healthy Controls	*p*
Number of vaccinated individuals	15	13	15	
Vaccination				0.711
Pfizer BioNTechBNT162b2, *n*	9	9	12	
Spikevax mRNA-1273, *n*	5	4	3	
Jcovden Ad26.COV2, *n*	1	0	0	
Booster dose (3rd dose) received, *n*	10	4	8	0.118
Days after vaccination, median (IQR)	167 (77)	148 (300)	215 (201)	0.156
Positive humoral response, *n* (%)	14 (93.3%)	13 (100%)	15 (100%)	1.000
Positive T-cell response *n*/yes	14 (93.3%)	12 (92.3%)	12 (80%)	0.596
Level of anti-S IgG, ratio, median (IQR)	5.4720 (4.08)	8.6260 (5.12)	9.4350 (2.13)	**0.035**
CD4+ cell response (INF-y) to S1 pool-specific protein, IU/mL, median (IQR)	0.4662 (0.86)	0.5845 (0.62)	0.2949 (0.70)	0.765
CD4+ and CD8+ cell response (INF-y) to S1 and S2 pool-specific proteins, IU/mL, median (IQR)	0.6451 (1.28)	0.5052 (0.92)	0.5503 (0.68)	0.966

Abbreviation: CVID—common variable immunodeficiency; sIgAD—selective IgA deficiency; *n*—number; IQR—interquartile range; mRNA—messenger ribonucleic acid; anti-S IgG—anti-spike mmunoglobulin G; INF-y—interferon-gamma; S1—S1 region of the severe acute respiratory syndrome coronavirus 2 spike glycoprotein; S2—S2 region of the severe acute respiratory syndrome coronavirus 2 spike glycoprotein. *p*-values of less than 0.05 were regarded as significant.

## Data Availability

The datasets generated during and/or analyzed during the current study are available from the corresponding author upon reasonable request.

## Supplementary Materials

The following supporting information can be downloaded at: https://www.mdpi.com/article/10.3390/vaccines11020354/s1, Supplementation S1: Gating strategy of B and T lymphocyte subsets.

## References

[1] Gathmann B., Grimbacher B., Beauté J., Dudoit Y., Mahlaoui N., Fischer A., Knerr V., Kindle G., Micol R., Benslama L. (2009). The European Internet-Based Patient and Research Database for Primary Immunodeficiencies: Results 2006–2008. Proceedings of the Clinical and Experimental Immunology.

[2] Yazdani R., Azizi G., Abolhassani H., Aghamohammadi A. (2017). Selective IgA Deficiency: Epidemiology, Pathogenesis, Clinical Phenotype, Diagnosis, Prognosis and Management. Scand. J. Immunol..

[3] Edwards E.S.J., Bosco J.J., Aui P.M., Stirling R.G., Cameron P.U., Chatelier J., Hore-Lacy F., O’Hehir R.E., van Zelm M.C. (2019). Predominantly Antibody-Deficient Patients With Non-Infectious Complications Have Reduced Naive B, Treg, Th17, and Tfh17 Cells. Front. Immunol..

[4] Lu H., Stratton C.W., Tang Y.-W. (2020). Outbreak of Pneumonia of Unknown Etiology in Wuhan, China: The Mystery and the Miracle. J. Med. Virol..

[5] Zhou P., Yang X.L., Wang X.G., Hu B., Zhang L., Zhang W., Si H.R., Zhu Y., Li B., Huang C.L. (2020). A Pneumonia Outbreak Associated with a New Coronavirus of Probable Bat Origin. Nature.

[6] Gao Y., Chen Y., Liu M., Shi S., Tian J. (2020). Impacts of Immunosuppression and Immunodeficiency on COVID-19: A Systematic Review and Meta-Analysis. J. Infect..

[7] Van der Made C.I., Netea M.G., van der Veerdonk F.L., Hoischen A. (2022). Clinical Implications of Host Genetic Variation and Susceptibility to Severe or Critical COVID-19. Genome Med..

[8] Liu B.M., Hill H.R. (2020). Role of Host Immune and Inflammatory Responses in COVID-19 Cases with Underlying Primary Immunodeficiency: A Review. J. Interferon Cytokine Res..

[9] Milito C., Lougaris V., Giardino G., Punziano A., Vultaggio A., Carrabba M., Cinetto F., Scarpa R., Delle Piane R.M., Baselli L. (2021). Clinical Outcome, Incidence, and SARS-CoV-2 Infection-Fatality Rates in Italian Patients with Inborn Errors of Immunity. J. Allergy Clin. Immunol. Pract..

[10] Bucciol G., Tangye S.G., Meyts I. (2021). Coronavirus Disease 2019 in Patients with Inborn Errors of Immunity: Lessons Learned. Curr. Opin. Pediatr..

[11] Katzenstein T.L., Rasmussen L.D., Drabe C.H., Larsen C.S., Hansen A.-B.E., Stærkind M., Knudsen L.S., Hansen C.H., Obel N. (2022). Outcome of SARS-CoV-2 Infection among Patients with Common Variable Immunodeficiency and a Matched Control Group: A Danish Nationwide Cohort Study. Front. Immunol..

[12] Tangye S.G., Bucciol G., Meyts I. (2021). Mechanisms Underlying Host Defense and Disease Pathology in Response to Severe Acute Respiratory Syndrome (SARS)-CoV2 Infection: Insights from Inborn Errors of Immunity. Curr. Opin. Allergy Clin. Immunol..

[13] Shields A.M., Burns S.O., Savic S., Richter A.G., Anantharachagan A., Arumugakani G., Baker K., Bahal S., Bermingham W., Bhole M. (2021). COVID-19 in Patients with Primary and Secondary Immunodeficiency: The United Kingdom Experience. J. Allergy Clin. Immunol..

[14] Goudouris E.S., Pinto-Mariz F., Mendonça L.O., Aranda C.S., Guimarães R.R., Kokron C., Barros M.T., Anísio F., Alonso M.L.O., Marcelino F. (2021). Outcome of SARS-CoV-2 Infection in 121 Patients with Inborn Errors of Immunity: A Cross-Sectional Study. J. Clin. Immunol..

[15] Kołtan S., Ziętkiewicz M., Grześk E., Becht R., Berdej-Szczot E., Cienkusz M., Ewertowska M., Heropolitańska-Pliszka E., Krysiak N., Lewandowicz-Uszyńska A. (2022). COVID-19 in Unvaccinated Patients with Inborn Errors of Immunity—Polish Experience. Front. Immunol..

[16] Çölkesen F., Kandemir B., Arslan Ş., Çölkesen F., Yıldız E., Korkmaz C., Vatansev H., Evcen R., Aykan F.S., Kılınç M. (2022). Relationship between Selective IgA Deficiency and COVID-19 Prognosis. Jpn. J. Infect. Dis..

[17] Naito Y., Takagi T., Yamamoto T., Watanabe S. (2020). Association between Selective IgA Deficiency and COVID-19. J. Clin. Biochem. Nutr..

[18] ESID-European Society for Immunodeficiencies. https://esid.org/COVID-19/ESID-COVID-19-Statement-March-2022.

[19] Pegu A., O’Connell S.E., Schmidt S.D., O’Dell S., Talana C.A., Lai L., Albert J., Anderson E., Bennett H., Corbett K.S. (2021). Durability of MRNA-1273 Vaccine–Induced Antibodies against SARS-CoV-2 Variants. Science.

[20] Post N., Eddy D., Huntley C., van Schalkwyk M.C.I., Shrotri M., Leeman D., Rigby S., Williams S.V., Bermingham W.H., Kellam P. (2020). Antibody Response to SARS-CoV-2 Infection in Humans: A Systematic Review. PLoS ONE.

[21] Widge A.T., Rouphael N.G., Jackson L.A., Anderson E.J., Roberts P.C., Makhene M., Chappell J.D., Denison M.R., Stevens L.J., Pruijssers A.J. (2021). Durability of Responses after SARS-CoV-2 MRNA-1273 Vaccination. N. Engl. J. Med..

[22] Long Q.X., Tang X.J., Shi Q.L., Li Q., Deng H.J., Yuan J., Hu J.L., Xu W., Zhang Y., Lv F.J. (2020). Clinical and Immunological Assessment of Asymptomatic SARS-CoV-2 Infections. Nat. Med..

[23] Gaebler C., Wang Z., Lorenzi J.C.C., Muecksch F., Finkin S., Tokuyama M., Cho A., Jankovic M., Schaefer-Babajew D., Oliveira T.Y. (2021). Evolution of Antibody Immunity to SARS-CoV-2. Nature.

[24] Shields A.M., Faustini S.E., Hill H.J., Al-Taei S., Tanner C., Ashford F., Workman S., Moreira F., Verma N., Wagg H. (2022). Increased Seroprevalence and Improved Antibody Responses Following Third Primary SARS-CoV-2 Immunisation: An Update From the COV-AD Study. Front. Immunol..

[25] Pham M.N., Murugesan K., Banaei N., Pinsky B.A., Tang M., Hoyte E., Lewis D.B., Gernez Y. (2022). Immunogenicity and Tolerability of COVID-19 Messenger RNA Vaccines in Primary Immunodeficiency Patients with Functional B-Cell Defects. J. Allergy Clin. Immunol..

[26] Shields A.M., Faustini S.E., Hill H.J., Al-Taei S., Tanner C., Ashford F., Workman S., Wagg H., Heritage G., Campton N. (2022). SARS-CoV-2 Vaccine Responses in Individuals with Antibody Deciency: Findings From The COV-AD Study upon Tyne Hospitals NHS Foundation Trust Teaching Hospitals NHS Trust. J. Clin. Immunol..

[27] Del Pino-Molina L., López-Granados E., Lecrevisse Q., Torres Canizales J., Pérez-Andrés M., Blanco E., Wentink M., Bonroy C., Nechvatalova J., Milota T. (2021). Dissection of the Pre-Germinal Center B-Cell Maturation Pathway in Common Variable Immunodeficiency Based on Standardized Flow Cytometric EuroFlow Tools. Front. Immunol..

[28] Amodio D., Ruggiero A., Sgrulletti M., Pighi C., Cotugno N., Medri C., Morrocchi E., Colagrossi L., Russo C., Zaffina S. (2021). Humoral and Cellular Response Following Vaccination With the BNT162b2 MRNA COVID-19 Vaccine in Patients Affected by Primary Immunodeficiencies. Front. Immunol..

[29] Sahin U., Muik A., Vogler I., Derhovanessian E., Kranz L.M., Vormehr M., Quandt J., Bidmon N., Ulges A., Baum A. (2021). BNT162b2 Vaccine Induces Neutralizing Antibodies and Poly-Specific T Cells in Humans. Nature.

[30] Tan A.T., Linster M., Tan C.W., Le Bert N., Chia W.N., Kunasegaran K., Zhuang Y., Tham C.Y.L., Chia A., Smith G.J.D. (2021). Early Induction of Functional SARS-CoV-2-Specific T Cells Associates with Rapid Viral Clearance and Mild Disease in COVID-19 Patients. Cell Rep..

[31] Sette A., Crotty S. (2021). Adaptive Immunity to SARS-CoV-2 and COVID-19. Cell.

[32] Le Bert N., Tan A.T., Kunasegaran K., Tham C.Y.L., Hafezi M., Chia A., Chng M.H.Y., Lin M., Tan N., Linster M. (2020). SARS-CoV-2-Specific T Cell Immunity in Cases of COVID-19 and SARS, and Uninfected Controls. Nature.

[33] Hagin D., Freund T., Navon M., Halperin T., Adir D., Marom R., Levi I., Benor S., Alcalay Y., Freund N.T. (2021). Immunogenicity of Pfizer-BioNTech COVID-19 Vaccine in Patients with Inborn Errors of Immunity. J. Allergy Clin. Immunol..

[34] Salinas A.F., Mortari E.P., Terreri S., Quintarelli C., Pulvirenti F., Di Cecca S., Guercio M., Milito C., Bonanni L., Auria S. (2021). SARS-CoV-2 Vaccine Induced Atypical Immune Responses in Antibody Defects: Everybody Does Their Best. J. Clin. Immunol..

[35] Delmonte O.M., Bergerson J.R.E., Burbelo P.D., Durkee-Shock J.R., Dobbs K., Bosticardo M., Keller M.D., McDermott D.H., Rao V.K., Dimitrova D. (2021). Antibody Responses to the SARS-CoV-2 Vaccine in Individuals with Various Inborn Errors of Immunity. J. Allergy Clin. Immunol..

[36] Shin J.J., Par-Young J., Unlu S., McNamara A., Park H.J., Shin M.S., Gee R.J., Doyle H., Afinogenova Y., Zidan E. (2022). Defining Clinical and Immunological Predictors of Poor Immune Responses to COVID-19 MRNA Vaccines in Patients with Primary Antibody Deficiency. J. Clin. Immunol..

[37] Ameratunga R., Longhurst H., Steele R., Lehnert K., Leung E., Brooks A.E.S., Woon S.T. (2021). Common Variable Immunodeficiency Disorders, T-Cell Responses to SARS-CoV-2 Vaccines, and the Risk of Chronic COVID-19. J. Allergy Clin. Immunol. Pract..

[38] Marshall J.C., Murthy S., Diaz J., Adhikari N., Angus D.C., Arabi Y.M., Baillie K., Bauer M., Berry S., Blackwood B. (2020). Personal View A Minimal Common Outcome Measure Set for COVID-19 Clinical Research. Lancet Infect. Dis..

[39] Tormo N., Navalpotro D., Martínez-Serrano M., Moreno M., Grosson F., Tur I., Guna M.R., Soriano P., Tornero A., Gimeno C. (2022). Commercial Interferon-Gamma Release Assay to Assess the Immune Response to First and Second Doses of MRNA Vaccine in Previously COVID-19 Infected versus Uninfected Individuals. Diagn. Microbiol. Infect. Dis..

[40] Chapel H., Lucas M., Lee M., Bjorkander J., Webster D., Grimbacher B., Fieschi C., Thon V., Abedi M.R., Hammarstrom L. (2008). Common Variable Immunodeficiency Disorders: Division into Distinct Clinical Phenotypes. Blood.

[41] Ameratunga R. (2018). Assessing Disease Severity in Common Variable Immunodeficiency Disorders (CVID) and CVID-Like Disorders. Front. Immunol..

[42] Piqueras B., Lavenu-Bombled C., Galicier L., Bergeron-Van Der Cruyssen F., Mouthon L., Chevret S., Debré P., Schmitt C., Oksenhendler E. (2003). Common Variable Immunodeficiency Patient Classification Based on Impaired B Cell Memory Differentiation Correlates with Clinical Aspects. J. Clin. Immunol..

[43] Warnatz K., Denz A., Dräger R., Braun M., Groth C., Wolff-Vorbeck G., Eibel H., Schlesier M., Peter H.H. (2002). Severe Deficiency of Switched Memory B Cells (CD27(+)IgM(-)IgD(-)) in Subgroups of Patients with Common Variable Immunodeficiency: A New Approach to Classify a Heterogeneous Disease. Blood.

[44] Wehr C., Kivioja T., Schmitt C., Ferry B., Witte T., Eren E., Vlkova M., Hernandez M., Detkova D., Bos P.R. (2008). The EUROclass Trial: Defining Subgroups in Common Variable Immunodeficiency. Blood.

[45] Driessen G.J., Van Zelm M.C., Van Hagen P.M., Hartwig N.G., Trip M., Warris A., De Vries E., Barendregt B.H., Pico I., Hop W. (2011). B-Cell Replication History and Somatic Hypermutation Status Identify Distinct Pathophysiologic Backgrounds in Common Variable Immunodeficiency. Blood.

[46] Prokofjeva T., Lucane Z., Kovalova Z., Kurjane N. (2022). Inborn Errors of Immunity in Latvia: Analysis of Data from 1994 to 2020. J. Clin. Immunol..

[47] Bergman P., Blennow O., Hansson L., Mielke S., Nowak P., Chen P., Söderdahl G., Österborg A., Smith C.I.E., Wullimann D. (2021). Safety and Efficacy of the MRNA BNT162b2 Vaccine against SARS-CoV-2 in Five Groups of Immunocompromised Patients and Healthy Controls in a Prospective Open-Label Clinical Trial. EBioMedicine.

[48] Squire J., Joshi A. (2021). Seroconversion after Coronavirus Disease 2019 Vaccination in Patients with Immune Deficiency. Ann. Allergy Asthma Immunol..

[49] Babaha F., Rezaei N. (2020). Primary Immunodeficiency Diseases in COVID-19 Pandemic: A Predisposing or Protective Factor?. Am. J. Med. Sci..

[50] Göschl L., Mrak D., Grabmeier-Pfistershammer K., Stiasny K., Haslacher H., Schneider L., Deimel T., Kartnig F., Tobudic S., Aletaha D. (2022). Reactogenicity and Immunogenicity of the Second COVID-19 Vaccination in Patients with Inborn Errors of Immunity or Mannan-Binding Lectin Deficiency. Front. Immunol..

[51] Pardi N., Hogan M.J., Porter F.W., Weissman D. (2018). MRNA Vaccines—A New Era in Vaccinology. Nat. Rev. Drug Discov..

[52] Friedmann D., Goldacker S., Peter H.H., Warnatz K. (2020). Preserved Cellular Immunity Upon Influenza Vaccination in Most Patients with Common Variable Immunodeficiency. J. Allergy Clin. Immunol. Pract..

[53] Dan J.M., Mateus J., Kato Y., Hastie K.M., Yu E.D., Faliti C.E., Grifoni A., Ramirez S.I., Haupt S., Frazier A. (2021). Immunological Memory to SARS-CoV-2 Assessed for up to 8 Months after Infection. Science.

[54] Ciabattini A., Nardini C., Santoro F., Garagnani P., Franceschi C., Medaglini D. (2018). Vaccination in the Elderly: The Challenge of Immune Changes with Aging. Semin. Immunol..

[55] Gardulf A., Abolhassani H., Gustafson R., Eriksson L.E., Hammarström L. (2018). Predictive Markers for Humoral Influenza Vaccine Response in Patients with Common Variable Immunodeficiency. J. Allergy Clin. Immunol..

[56] Beck C.R., McKenzie B.C., Hashim A.B., Harris R.C., Nguyen-Van-Tam J.S. (2012). Influenza Vaccination for Immunocompromised Patients: Systematic Review and Meta-Analysis by Etiology. J. Infect. Dis..

