# Supplementary material for: Long-Term Immunological Memory of SARS-CoV-2 Is Present in Patients with Primary Antibody Deficiencies for up to a Year after Vaccination
Source: Vaccines (Basel). 2023 Feb 3;11(2):354. doi: 10.3390/vaccines11020354 (PMC9959530; doi:10.3390/vaccines11020354)

Supplement S1. Gating strategy of B and T lymphocyte subsets.

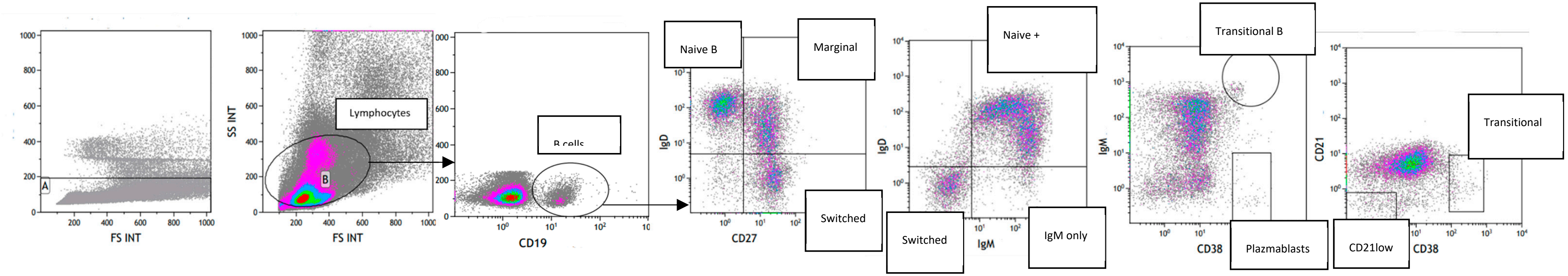

A B cell gating for immonophenotyping classification systems (Paris, Freiburg, EUROClass, B-cell patern).

B B cell gating

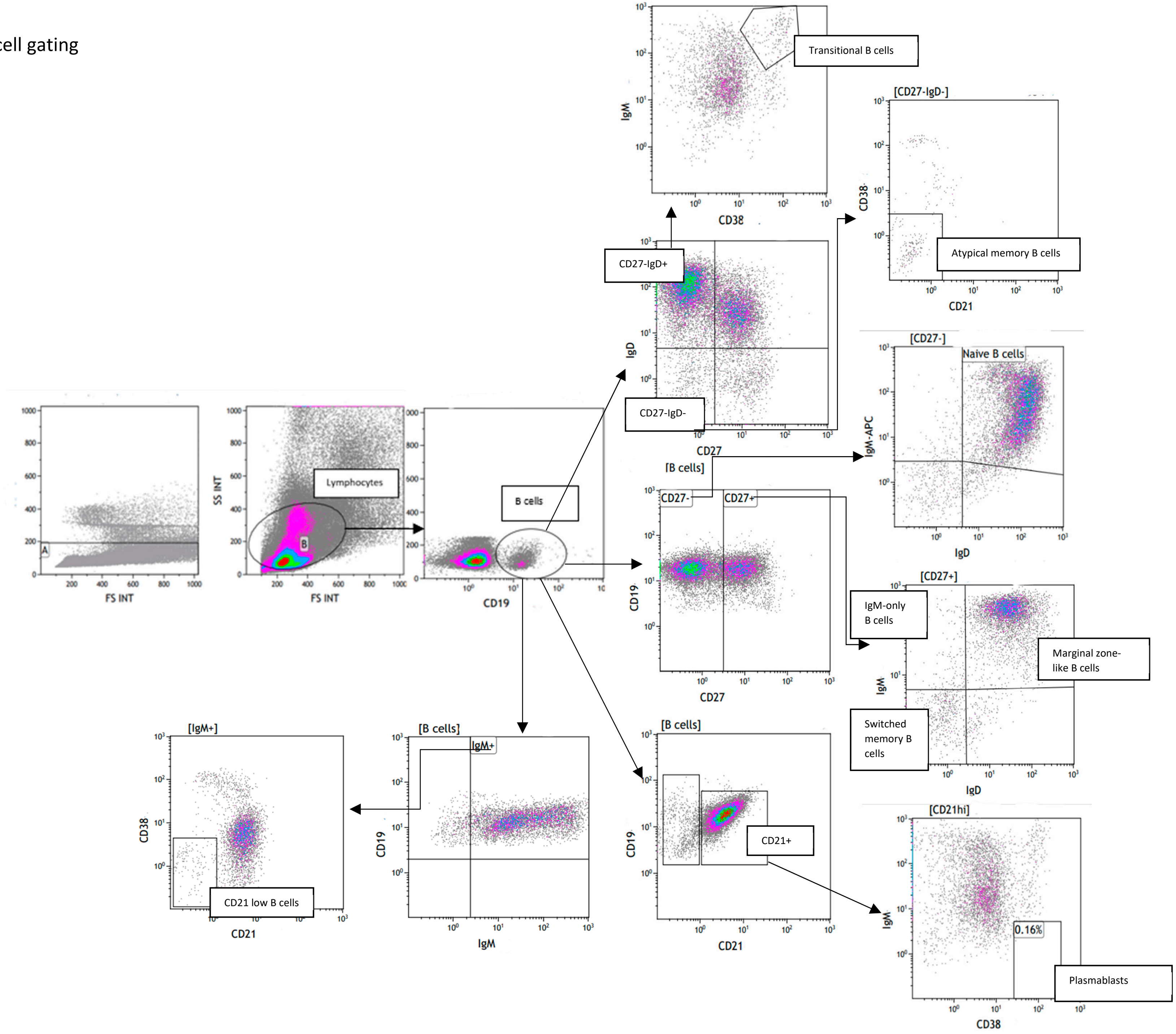

C T cell gating

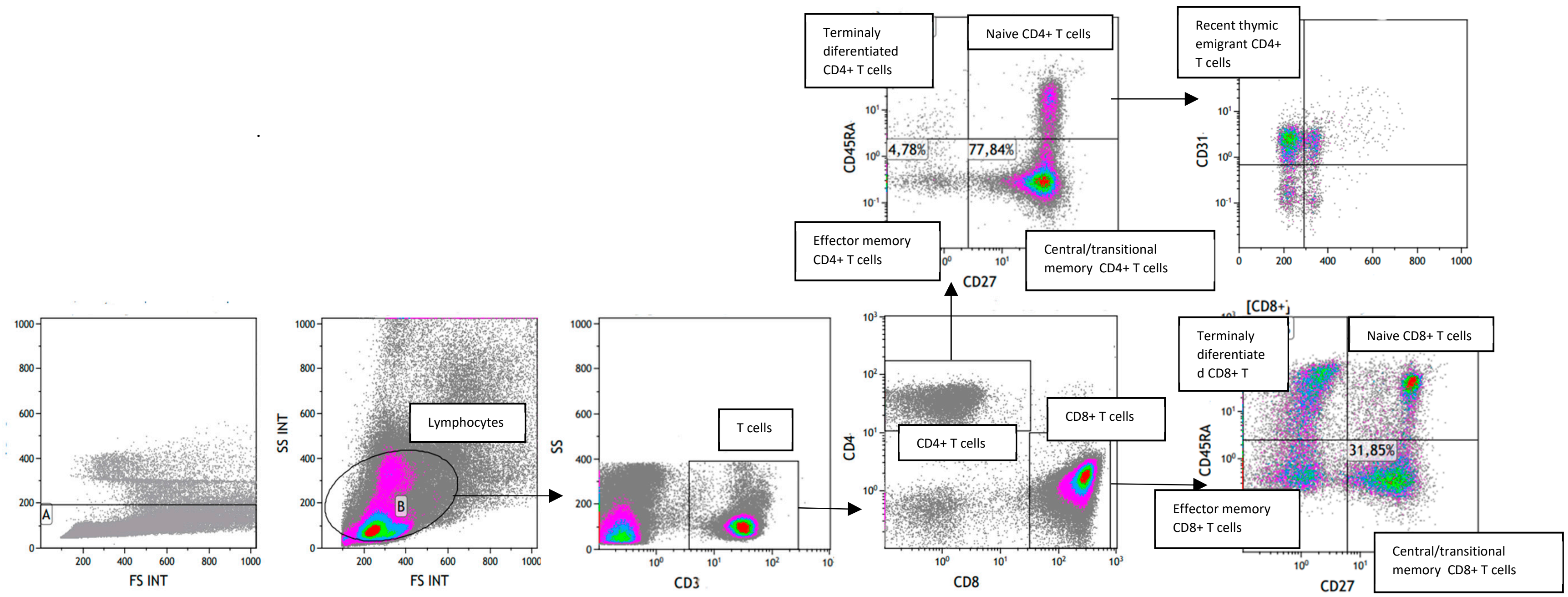

D T regulatory cell gating

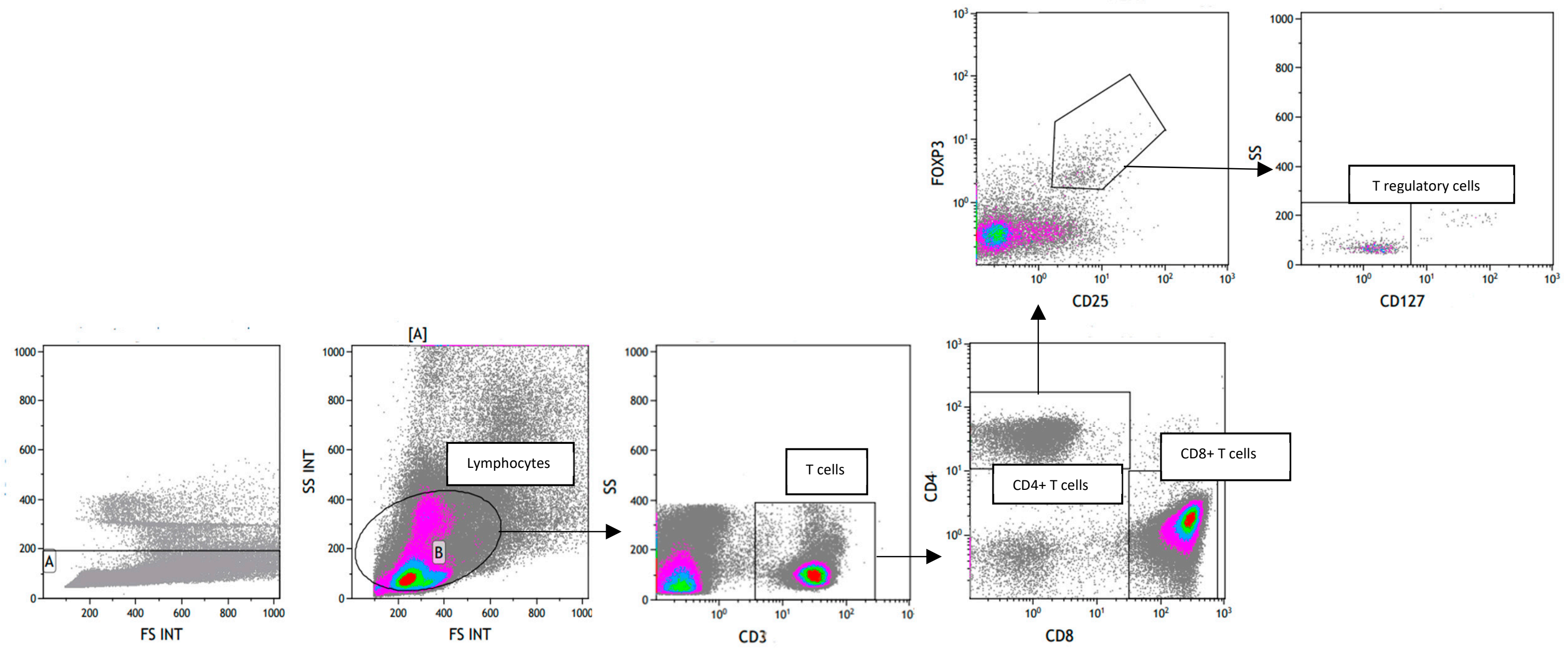

Supplement: Supplementary file 1 [file vaccines-11-00354-s001.zip › vaccines-2170611-supplementary.pdf]
